# Supplementary material for: ETS1 Protein Expression May Be Altered by the Complementarity of ETS1 mRNA Sequences with miR-203a-3p and miR-204-3p in Papillary Thyroid Carcinoma
Source: Int J Mol Sci. 2025 Jan 31;26(3):1253. doi: 10.3390/ijms26031253 (PMC11818258; doi:10.3390/ijms26031253)
Supplement: Supplementary file 1 [file ijms-26-01253-s001.zip › ijms-3423806-supplementary.pdf]

Table S1: Comparison of ETS1 expression in divergent variants of PTC by cell compartments

Test	Compared Sample	Subcellular compartment					
		Nucleus		Cytoplasm		Total	
		p	Adj. p^a	*p*	Adj. p^a	*p*	Adj. p^a
Median	All 4 PTC subtypes	**0.016**	/	0.773	/	0.859	/
Pairwise Comparisons (for median values)	cv – mix	0.547	1.000				
	rare - mix	0.269	1.000				
	fv – mix	0.026	0.157				
	cv - rare	0.045	0.271				
	cv - fv	0.005	**0.027**				
	fv -rare	0.072	0.432				
Kruskal-Wallis	All 4 PTC subtypes	**0.017**	/	0.853	/	0.507	/
Pairwise Comparisons (for distribution)	cv – mix	0.840	1.000				
	rare - mix	0.415	1.000				
	fv – mix	0.009	**0.054**				
	cv - rare	0.537	1.000				
	cv - fv	0.015	0.089				
	fv -rare	0.062	0.372				

p: asymptotic significances (2-sided tests) are displayed. Significant *p*-values are bolded. Adj. p^a: adjusted *p*-value. Significance values have been adjusted by the Bonferroni correction for multiple tests. Total: nucleus + cytoplasm. PTC: papillary thyroid carcinoma.
cv: classical variant of PTC, fv: follicular variant of PTC, mix: mixed classical and follicular variant of PTC, rare: rare variants of PTC.

Table S2: Comparison of ETS1 expression in divergent nonmalignant thyroid neoplasia by cell compartments

Test	Compared Sample	Subcellular compartment					
		Nucleus		Cytoplasm		Total	
		p	Adj. p^a	*p*	Adj. p^a	*p*	Adj. p^a
Median	All 4 NMT subtypes	**0.000**	/	**0.001**	/	**0.000**	/
Pairwise Comparisons (for median values)	NTT-NG	0.047	0.280	0.000	**0.000**	0.047	0.280
	NTT-TA	0.000	**0.002**	0.003	**0.015**	0.000	**0.002**
	NTT-Thy	0.195	1.000	0.001	**0.007**	0.195	1.000
	NG-Thy	0.639	1.000	0.210	1.000	0.639	1.000
	TA-Thy	0.629	1.000	0.783	1.000	0.629	1.000
	NG-TA	0.973	1.000	0.292	1.000	0.682	1.000
Kruskal-Wallis	All 4 NMT subtypes	**0.004**	/	**0.001**	/	**0.003**	/
Pairwise Comparisons (for distribution)	NTT-Thy	0.049	0.493	0.055	0.549	0.074	0.743
	NTT-NG	0.009	0.086	0.000	**0.002**	0.004	**0.041**
	NTT-TA	0.001	**0.014**	0.034	0.338	0.001	**0.014**
	NG-Thy	0.892	1.000	0.269	1.000	0.631	1.000
	TA-Thy	0.505	1.000	0.919	1.000	0.411	1.000
	NG-FT	0.528	1.000	0.168	1.000	0.674	1.000

p: asymptotic significances (2-sided tests) are displayed. Significant *p*-values are bolded. Adj. p^a: adjusted *p*-value. Significance values have been adjusted by the Bonferroni correction for multiple tests. Total: nucleus + cytoplasm. NTT: normal (healthy) thyroid tissue, Thy: thyroiditis, NG: nodular goiter, TA: thyroid adenoma.
